# Evaluation of clinical and laboratory characteristics of dengue viral infection and risk factors of dengue hemorrhagic fever: a multi-center retrospective analysis

**DOI:** 10.1186/s12879-024-09384-z

**Published:** 2024-05-17

**Authors:** Muhammad Riaz, Sabriah Noor Binti Harun, Tauqeer Hussain Mallhi, Yusra Habib Khan, Muhammad Hammad Butt, Aamir Husain, Muhammad Mujeeb khan, Amer Hayat Khan

**Affiliations:** 1 Discipline of Clinical Pharmacy, School of Pharmaceutical Sciences, University Sains Malaysia, Palau Penang, Malaysia; 2 Faculty of Pharmaceutical Sciences, Government College University Faisalabad, Faisalabad, Pakistan; 3 Department of Clinical Pharmacy, College of Pharmacy, Jouf University, Sakakah, Al-Jouf Kingdom of Saudi Arabia; 4 Department of Medicinal Chemistry, Faculty of Pharmacy, Uppsala University, Uppsala, 75123 Sweden; 5 Department of Medicine, Faisalabad Medical University, Faisalabad, Pakistan; 6 Department of Infectious Diseases, Rawalpindi Medical University, Rawalpindi, Pakistan

**Keywords:** Dengue, Dengue fever, Dengue hemorrhagic fever, Pakistan, Severe dengue, Mortality

## Abstract

**Background:**

Dengue Viral Infection (DVI) has become endemic in Pakistan since the first major outbreak in Karachi in 1996. Despite aggressive measures taken by relevant authorities, Pakistan has been dealing with a worsening dengue crisis for the past two decades. DHF is severe form of dengue infection which is linked with significant morbidity and mortality. Early identification of severe dengue infections can reduce the morbidity and mortality. In this context we planned current study in which we find out the different factors related with DHF as well as clinical laboratory features of DHF and compare them to DF so that patients can be best evaluated for DHF and managed accordingly at admission.

**Methods:**

Retrospective study conducted over a period of 6 years (2013–2018) in two tertiary care hospitals in Pakistan. Data were collected by using a pre-structured data collection form. Data were statistically analyzed to determine the clinical and laboratory characteristics of DVI and risk factors of dengue hemorrhagic fever (DHF).

**Results:**

A total 512 dengue cases (34.05 ± 15.08 years; Male 69.53%) were reviewed. Most common clinical manifestations of DVI were fever (99.60%), headache (89.1%), chills (86.5%), rigors (86.5%), myalgia (72.3%). Less common clinical manifestations were vomiting (52.5%), arthralgia (50.2%) and skin rashes (47.5%). Furthermore, nasal bleeding (44.1%), gum bleeding (32.6%), pleural effusion (13.9%) and hematuria (13.1%) were more profound clinical presentations among DHF patients. Mortality rate was 1.5% in this study. Logistic regression analysis indicated that delayed hospitalization (OR: 2.30) and diabetes mellitus (OR:2.71), shortness of breath (OR:2.21), association with risk groups i.e., living near stagnant water, travelling to endemic areas, living in endemic regions (OR:1.95), and presence of warning signs (OR:2.18) were identified as risk factors of DHF. Statistically we found that there is strong association of diabetes mellitus (DM) with DHF while the patient suffering from DM individually had higher odds (2.71) of developing DHF than patients without disease.

**Conclusions:**

The current study demonstrated that the clinical and laboratory profiles of DF and DHF are significantly distinct. Significant predictors of DHF were advanced age, diabetes mellitus, ascites, pleural effusion, thick gallbladder and delayed hospitalization. The identification of these factors at early stage provides opportunities for the clinicians to identify high risk patients and to reduce dengue-related morbidity and mortality.

## Introduction

Dengue Viral Infection (DVI) is a mosquito-borne viral disease and considered a global health problem. The global burden of dengue virus infection is 390 million cases reported every year [1]. The incidence of DVI has increased 30 times during the last 50 years [2]. DVI is classified as classical dengue fever (DF), non-classical DF, dengue hemorrhagic fever (DHF), and dengue shock syndrome (DSS) [3]. The global trend of increasing DVI cases has also increased its burden in Pakistan. Due to the condensed inter-epidemic phase, Pakistan experiences a dengue epidemic nearly every year or every other year. Despite government efforts, disease relapses persist, resulting in an increase in disease severity and mortality.

Pakistan has diverse climates, poor health care infrastructure, lack of hygienic measures, poor housekeeping, lack of mosquito control measures, rapid urbanization, and increased international travel in Pakistan. These factors create ideal conditions for DVI transmission in the country [4–6]. Pakistan has reported more severe dengue cases due to condensation in inter-epidemic periods [3, 5–7]. According to the National Institute of Health (NIH) Islamabad, the number of cases reported in Pakistan in 2017, 2018, 2019, and 2020 were 22,938, more than 3,200, 24,547, and 3,442, respectively. In 2020, only 1,153 cases were reported during a comparable time period. By the end of November 2021, 48,906 cases have been reported in the country. By the end of November 2022, 75,450 cases were reported in Pakistan with highest number from KPK. Between November 2021 and December 2021, there were 16,388 reported cases [8].

The DHF, the severe form of dengue, is currently the most debilitating and life-threatening because of its fatal outcome. The severe infections can be managed effectively by identifying their risk factors. However, there are dearth of investigations evaluating risk factors of DHF in Pakistan. Other studies conducted in Pakistan focus on the clinical profile of the dengue infection [9], have small sample size [5, 10], clinical and laboratory features of the disease [7], association of DVI with thrombocytopenia and leucopenia [8], dengue serotypes [11], and detection of DVI using immunoglobulins and nonstructural protein 1 (NS-1) antigens [12].

Since prioritized management of severe dengue can reduce morbidity and mortality, identifying risk factors will help with optimal care, aid in the prioritization of resources for those at high risk of severe disease and assist scientists better understanding disease dynamics. Understanding the distinctive clinical and laboratory features of various severities of DVI facilitate more informed decisions, as well as developing effective management strategies. Give the paucity of data in Pakistan, this multicenter retrospective study was conducted to elucidate the clinic-laboratory features of DVI and risk factors of DHF.

## Methods

### Ethics statement

This study is approved by research and ethical committee of Holy Family Hospital Rawalpindi Pakistan (235/IRER/RMU/2020) and ethical review committee of Allied Hospital Faisalabad Pakistan (FMU/registration No.910), the two study sites where the data were collected. All data was analyzed anonymously; the data was collected retrospectively, so the research and ethical committee of Holy Family Hospital Rawalpindi Pakistan and ethical review committee of Allied Hospital Faisalabad Pakistan waived the requirement of informed consents. The patients were identified using their hospital/medical registration number (RN) at their respective record centers. Before data analysis, case information was retrieved and each case was assigned a unique numerical code.

### Study location

This study was conducted in two hospitals; Allied hospital Faisalabad and Holy Family Hospital Rawalpindi, having 1150 and 850 beds, respectively. Allied hospital serves 2.5–3.0 million residents of Faisalabad, a third largest city of Pakistan. Holy Family hospital Rawalpindi serve 1.5–2.0 million residents of Rawalpindi and also serves as referral center for nearby states [13, 14].

### Study population

The target population of the purpose of the current study was patients with DVI. All the patients with confirmed diagnosis of DVI admitted during 6 years period (2013–2018) were included. Although, all suspected cases were taken into the study but cases meeting only inclusion and exclusion criteria were selected for analysis.

### Inclusion and exclusion criteria

Patients with all ages, confirmed diagnosis of dengue by ELISA, RT-PCR or HAI, with complete laboratory data RFTs, LFTs, CBCs, and demographics at admission as well as at discharge were included in this study. On the other hand, patients with confirmed diagnosis of dengue but incomplete data on demographics and laboratory parameters were excluded from the study, as this data were needed to evaluate the primary objectives of the current study.

### Dengue diagnosis and classification

**Fig. 1:**
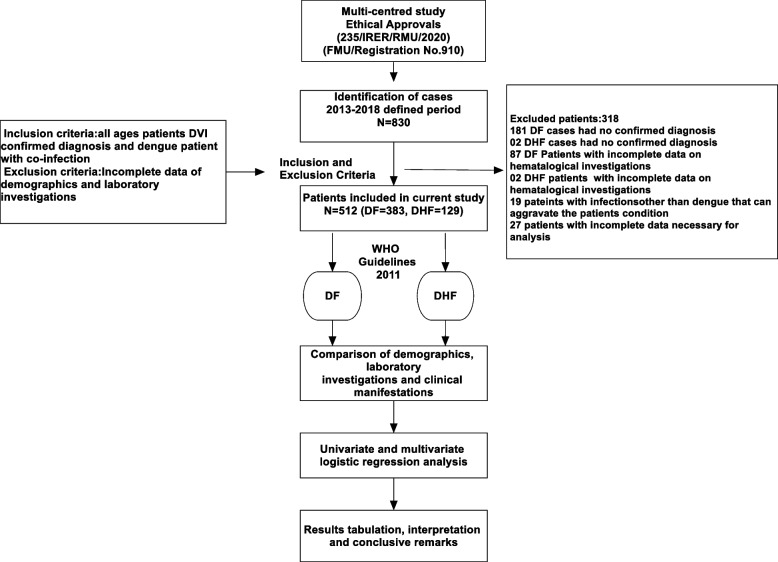
Study flow diagram

Clinical case definition of dengue viral infection is suspected dengue infection was defined as the presence of fever and any two of the below mentioned symptoms fever, myalgia, arthralgia, headache, skin rash, retro-orbital pain, hemorrhagic manifestation(s) [15]. Probable dengue infection was defined as the presence of low in white blood cell count ˂ 4000 and low in platelet cells count ˂ 100,000 [16] or hematocrit ˂ 30% or ˃ 55%. Confirmed case for the dengue viral infection was defined as positive NS-1 or viral detection by PCR or seroconversion from negative for dengue virus specific IgM antibody in acute phase (˂ 5days after onset of symptoms) to positive for dengue virus specific IgM antibody in convalescent phase specimen collected ≥ 5 days after onset of symptoms or ≥ 4-fold rise in titer of IgG in paired acute and convalescent serum sample [17]. Dengue diagnosis was confirmed by direct method (RT-PCR/ELIZA/NS-1) and indirect method (IgM and IgG antibodies in the serum). These methods were used for the confirmation of suspected cases by using at least one of the following criteria: (1) Positive reverse transcriptase polymerase chain reaction (RT-PCR) result, (2) presence of dengue specific IgM and IgG anti-bodies in acute phase serum by enzyme linked immunosorbent Cal biotech Inc. ELISA test system (Catalog No. DE051M) [14, 18]. The serum samples were also tested for dengue-specific NS-I (pan-E Early dengue ELISA kit by Panbio, Australia and Platelia dengue NS1Ag assay by Bio-Rad Laboratories, USA). Only confirmed dengue cases were included in analysis. Primary dengue infection was distinguished from secondary infection by using IgM-IgG ratio where dengue infection was defined as primary if ratio ≥ 1.8 and as secondary if < 1.8 [19–21]. Serologically confirmed dengue patients were subjected to clinical case definition and disease severity and were further classified into DF, DHF and DSS according to WHO guidelines 2011 [17] and other studies conducted elsewhere use the similar criteria [18, 22]. DF is defined as presence of fever and two or more of the following, retro-orital or ocular pain, rash, myalgia, arthralgia, leukopenia, or hemorrhagic manifestations but not meeting the case definition of dengue hemorrhagic fever. DHF is characterized by evidence of plasma leakage as shown by hemoconcentration (increase in hematocrit ≥ 20% or decrease in hematocrit ≥ 20% of baseline following fluid replacement therapy) or pleural effusion or ascites or hyponatremia. DSS has all the criteria for DHF plus circulatory failure as evidence by rapid and weak pulse and narrow pulse pressure (˂ 20mmHg) or age-specific hypotension and cold, clammy skin, and restlessness. This classification system is primarily used for the management of disease in Pakistan. Study methodology with patient’s inclusion and exclusion criteria shown in Fig. 1.

### Data collection form

Patients were identified based on their respective record centers, and data were extracted from patient record files utilizing a pre-structured data collection form. The data collection form included sections for demographics (age, weight, socioeconomic status, gender, residence in rural and urban areas), diagnostic techniques (RT-PCR/ELIZA/NS-1 and IgM, IgG antibodies), family and disease history (kidney diseases, Hypertension, diabetes and liver diseases), clinical presentations ( Fever ˃ 2 and ˂ 10 days and associated two symptoms from headache, retro-orbital pain, myalgia, bone pain), warning signs includes persistent vomiting, lethargy, restlessness, increase in bleeding tendencies, severe abdominal pain and laboratory results ( CBC, INR, PT and APTT, LFTs, RFTs). The laboratory results were documented both at the time of admission and at the time of discharge. Patients’ information was collected for each day of hospitalization until discharge or death, whichever occurred first.

### Operational definitions

Hospital stay: is defined by ≥ 1 day bed occupancy in hospital, Severe Dengue: refers to presence of DHF and DSS, Thrombocytopenia: platelets count < 150,000/µL cells. Elevated transaminases: elevation of liver enzymes such as AST and/or ALT > 2 times the normal value, Transaminitis: elevation of both ALT and AST. Multiple organ dysfunctions (MODs): refer to dysfunction of two or more organs, Diabetes mellitus (DM): Repeated determination of fasting plasma glucose level (> 110 mg/dL) or prescription of anti-diabetic medications, Hypertension: An average systolic/diastolic blood pressure of 140/90mmHg or prescription of anti-hypertensive medications Prolonged PT: PT > 15 s, Prolonged aPTT: aPTT > 35 s, Prolonged hospital stay: Hospital stay greater than 3 days, Delayed Hospitalization: Hospital admission after day five of onset of illness was referred to delayed or late hospitalization, Warning signs: Clinical fluid accumulation (Ascites, pleural effusion), Liver enlargement > 2 cm, severe abdominal pain or tenderness, persistent vomiting (at least three episodes/24 h), mucosal bleed, lethargy or restlessness rapid decline in platelets count with concurrent increase in hematocrit. Dengue risk groups: Family history of dengue, living in non-fogging zone living near stagnant water resources or construction sites, travelling to jungle. While focusing on these definitions and clinical laboratory spectrum patients were divided into DF and DHF for the purpose of comparison in analysis.

### Statistical analysis

Statistical Analysis was performed using SPSS software version 25. Continuous variables were recorded as means and standard deviations (SD) while categorical variables were recorded as frequencies and percentages while unless otherwise stated. Categorical and continuous variables were analyzed using Chi-Square or Fischer-Exact test and independent *t*-test respectively. Logistic regression model was performed to determine the factors independently associated with severe form of dengue infection (DHF). The variables with *P* values less than 0.25 in univariate were considered as candidates for multivariate analysis. The use of univariate *P* values < 0.25 has advantage of tending to include more variables in multivariate analysis while traditional levels of *P* value such as 0.05 can fail in identifying variables known to be important [23]. Descriptive value below 0.005 was considered statistically significant.

## Results

Of 830 DVI suspected cases assessed for the purpose of this study, 512 cases that met the inclusion criteria were included into the final analysis (Fig. 1). Of 318 excluded cases, majority of them (*n* = 183) had no confirmed diagnosis for DVI. The details of other cases excluded from this study are provided in Fig. 1.

The mean age of patients was 34.05 ± 15.08 years. There was unequal distribution between male to female, where more males were in this study (Table 1). Majority of the population comprised of adults and belong to urban areas. Socioeconomic status includes low (13.09%) middle (82.82%) and high (4.69%) income categories. The studied patients were also evaluated for their risks of contracting DVI, which included living in dengue endemic areas (32.81%), traveling to dengue endemic areas (25.58%), residing near stagnant water sources (16.01%) or mosquito production site (12.89%), living in non- fogging zone (3.71%) or mega structure (1.76%) and having a family history of DVI (5.28%).

A total eight fatal cases (1.6%) were observed in this study (DF:6, DHF:2). Age > 40 years was in 7 (87.5%) patients and warning signs were observed in 2 (0.25%) fatal cases. All of these cases were admitted to the hospitals on third day of illness. Two fatal cases had MODs, three had shock, three had respiratory failure, while all eight cases had renal complications.

**Table 1:** Comparison of demographics and clinical features between the DF and DHF

Variable	Total Cases (*N* = 512) n (%)	DF (*N* = 383) n (%)	DHF (*N* = 129) n (%)	*P-value
**Age**				
Age (years)^a^	34.1 ± 15.1	33.1 ± 15.4	37.0 ± 16.8	**0.014**
Age ≤ 40	382 (77.5)	297 (47.5)	85 (22.2)	**0.010**
Age > 40	130 (25.4)	86 (22.5)	44 (34.1)	
**Gender**				
Female	156 (30.5)	113 (29.5)	43 (33.4)	0.439
Male	356 (69.5)	270 (70.5)	86 (66.7)	
**Residence**				
Rural	63 (12.3)	41 (10.7)	22 (17.1)	0.058
Urban	449 (89.7)	342 (89.3)	107 (82.9)	
**Socioeconomic status**				
Low (˂ 20 K)^b^	67 (13.1)	18 (14.0)	49 (12.8)	0.937
Middle (20–40 K)	424 (82.9)	106 (82.2)	318 (83.0)	
High (˃40 K)	21 (4.1)	5 (3.9)	16 (4.2)	
**Dual infection**				
Yes	174 (33.1)	129 (33.7)	45 (34.9)	0.803
No	338 (66.0)	254 (66.3)	84 (65.1)	
**Co-morbidities**				
COPD	2 (0.4)	1 (0.3)	1 (0.8)	0.481
Asthma	9 (1.8)	6 (1.6)	3 (2.3)	0.570
HTN	78 (15.2)	52 (13.6)	26 (20.2)	0.072
CHF	5 (0.1)	3 (0.8)	2 (1.6)	0.576
IHD	14 (2.7)	11 (2.9)	3 (2.3)	0.742
RA	5 (0.1)	4 (1.0)	1 (0.8)	0.788
Osteoporosis	1 (0.2)	00	1 (0.1)	0.085
DM	44 (8.5)	23 (6.01)	21 (16.3)	**< 0.001**
Liver cirrhosis	6 (0.1)	2 (0.5)	4 (3.1)	**0.016**
Hyperlipidemia	34 (6.6)	29 (7.6)	5 (3.9)	0.202
CKD	27 (5.3)	11 (2.9)	16 (12.4)	**< 0.001**
Association with risk group	167 (32.6)	106 (27.7)	61 (47.3)	**< 0.001**
Secondary infection	7 (1.4)	6 (1.6)	1 (0.8)	0.503
Warning sings	125 (24.4)	60 (15.7)	65 (50.4)	**< 0.001**
Delayed hospitalization	125 (31.8)	121 (31.7)	42 (32.6)	0.142
Days of illness before admission	4.7 ± 2.3	4.7 ± 2.3	4.8 ± 2.2	0.919
Stay days	4.1 ± 2.2	3.9 ± 1.6	4.7 ± 3.4	**0.002**
Duration of fever	2.2 ± 1.3	2.1 ± 1.2	2.4 ± 1.4	0.086
Mortality	8 (1.6)	6 (1.5)	2 (1.6)	0.982

*Abbreviations*: *COPD* Chronic obstructive pulmonary disease, *HTN* Hypertension, *CHF* Congestive heart failure, *IHD* Ischemic heart disease, *RA* Rheumatoid arthritis, *DM* Diabetes Mellitus

*Fisher’s exact test (if more than 20% of cells with expected counts of less than 5) while all the other *P* values were calculated by pears on Chi-square

^a^Age calculated as a mean ± standard deviation

^b^Household income in thousands

Based on DVI severity classification, DF was present in 74.80% (*n* = 383/512) of the study population, while DHF was observed in 25.1% (*n* = 129/512) of patients. NAAT test for antibody detection (IgM, IgG, or both) and NS-I antigen by ELISA technique were used in 88.3% (*n* = 452/512) and 85.1% (*n* = 436/512), respectively, for the detection of DVI and its serotypes. Our analysis ascertained that patient having age > 40 years (34.1%), diabetes mellitus (16.3%), CKD (12.4%) and warning signs (50.4%) were more likely to have severe dengue infection (DHF/DSS). Patients with DHF had significantly prolonged hospital stay than those with DF (Table 1).

Patients were presented with the common clinical manifestations such as fever (99.60%), headache (89.1%), chills (86.5%), rigors (86.5%), myalgia (72.3%). Less common clinical manifestations were vomiting (52.5%), arthralgia (50.2%) and skin rashes (47.5%) (Table 2). The prevalence of lethargy, nasal and gum bleeding, diarrhea, thick gall bladder, sore throat, ascites, and pleural effusion was significantly higher among patients with DHF compared to those with DF.

**Table 2:** Comparison of clinical manifestation between the patients with DF and DHF

Clinical manifestation	Total cases (*N* = 512) n (%)	DF (*N* = 383) n (%)	DHF (*N* = 129) n (%)	P*-value
Fever	510 (99.6)	303 (79.1)	102 (79.1)	1.00
Headache	405 (89.1)	303 (79.1)	102 (79.1)	1.00
Chills	443 (86.5)	336 (94.8)	107 (82.1)	0.169
Rigors	443 (86.5)	341 (89.0)	102 (79.1)	**0.004**
Myalgia	370 (72.3)	183 (49.5)	87 (67.4)	0.157
Vomiting	269 (52.5)	197 (51.4)	72 (55.8)	0.416
Arthralgia	258 (50.2)	193 (50.4)	65 (50.4)	0.099
Nausea	261 (50.1)	202 (52.7)	59 (45.7)	0.186
Skin rashes	244 (47.5)	188 (49.1)	56 (43.4)	0.308
Splenomegaly	162 (31.6)	123 (32.1)	39 (30.2)	0.743
Retro-orbital pain	149 (29.1)	62 (16.2)	87 (67.4)	0.491
Lethargy	144 (28.1)	97 (25.3)	47 (36.4)	**0.018**
Restlessness	124 (24.2)	88 (22.1)	36 (27.9)	0.258
Hepatomegaly	126 (24.6)	88 (22.1)	38 (29.5)	0.139
Anorexia	112 (21.9)	81 (21.2)	31 (24.0)	0.538
Abdominal pain	109 (21.3)	74 (19.3)	35 (27.1)	0.063
Dizziness	97 (18.1)	75 (19.6)	22 (17.1)	0.526
Shortness of breath	79 (15.4)	47 (12.3)	32 (24.8)	**0.001**
Dehydration	71 (13.9)	60 (15.7)	11 (8.5)	**0.042**
Nasal bleeding	70 (13.7)	12 (3.1)	58 (44.1)	**< 0.001**
Gum bleeding	60 (11.7)	18 (4.7)	42 (32.6)	**< 0.001**
Diarrhea	52 (10.2)	30 (7.8)	22 (17.1)	**0.003**
Malaise	47 (9.2)	33 (8.6)	14 (10.9)	0.447
Thick gall bladder	40 (7.8)	24 (6.3)	16 (12.4)	**0.025**
Sore throat	38 (7.4)	27 (7.0)	11 (8.5)	**< 0.001**
Ascites	37 (7.2)	16 (4.2)	21 (16.2)	**< 0.001**
Pleural effusion	33 (6.5)	15 (3.9)	18 (13.9)	**< 0.001**
Hematuria	27 (5.3)	9 (2.3)	18 (13.1)	< **0.001**
Chest pain	18 (3.5)	14 (3.7)	4 (3.1)	0.767
Flushing	14 (2.7)	11 (2.9)	3 (2.3)	0.742
Petechiae	12 (2.3)	1 (0.3)	11 (8.5)	0.767
Blood in stool	10 (1.9)	1 (0.3)	9 (6.1)	**< 0.001**
Jaundice	9 (1.8)	4 (1.0)	5 (3.9)	**0.034**
Vertigo	6 (1.2)	6 (1.6)	0 (0)	0.153
Hemoptysis	10 (1.1)	2 (0.5 )	8 (6.2)	**< 0.001**
Less oral intake	5 (1.0)	3 (0.8)	2 (1.6)	0.444
Purpura	2 (0.4)	2 (0.5)	0 (0)	1.00
Palpitations	2 (0.4)	2 (0.5)	0 (0)	1.00
Anasarca	2 (0.4)	0 (0)	2 (1.6)	0.063
Lymphadenopathy	2 (0.4)	2 (0.5)	0 (0)	1.00
Metorrhagia	1 (0.2)	1 (0.3)	0 (0)	0.560

*Fisher’s exact test (if more than 20% of cells with expected counts of less than 5) while all the other *P* values were calculated by pears on Chi-square

The comparison of laboratory characteristics between DF and DHF showed that the serum creatinine, hematocrit, aPTT and PT were significantly higher among patients with DHF as compared to DF (Table 3). The levels of platelets, WBCs and hemoglobin were also significantly lower among patient with DHF than DF.

**Table 3:** Comparison of clinical laboratory features in patients with DF and DHF on admission

Variable	Total cases (512) Mean ± SD	DF (383) Mean ± SD	DHF (129) Mean ± SD	*P-value
Fever (ºC)	99.6 ± 1.8	99.6 ± 1.9	99.6 ± 1.5	0.983
Pulse rate (BPM)	80.6 ± 17.3	79.1 ± 17.5	82.4 ± 16.4	0.173
Serum Cr (mg/dL)	1.3 ± 3.5	1.3 ± 3.9	1.4 ± 1.9	**< 0.001**
GFR by EPI (mL/min)	89.5 ± 32.4	92.3 ± 31.5	80.9 ± 33.8	**< 0.001**
GFR by MDRD (mL/min)	87.7 ± 36.9	90.4 ± 34.4	79.6 ± 42.6	**< 0.001**
Total protein (g/L)	6.8 ± 0.9	6.7 ± 0.9	6.1 ± 0.9	**< 0.001**
Albumin (mg/L)	3.1 ± 0.8	3.1 ± 0.9	3.1 ± 0.7	**< 0.001**
Globulin (mg/L)	2.4 ± 0.7	2.4 ± 0.7	2.5 ± 0.7	**0.001**
Total bilirubin (mg/dL)	0.8 ± 0.6	1.2 ± 1.8	1.1 ± 1.1	0.359
Hemoglobin (mg/dL)	13.4 ± 8.1	13.7 ± 9.2	12.7 ± 9.2	**0.001**
Hematocrit (%)	38.6 ± 6.9	38.6 ± 6.7	42.3 ± 7.5	**0.002**
PT (sec)	16.2 ± 3.3	15.2 ± 2.4	19.1 ± 2.4	**< 0.001**
APTT (sec)	38.4 ± 9.9	33.6 ± 3.4	52.6 ± 9.6	**< 0.001**
INR (sec)	0.9 ± 0.2	0.9 ± 0.1	1.2 ± 0.4	0.185
Thrombocytopenia	398 (77.7)	292 (76.2)	106 (82.2)	0.068
AKI	152 (29.7)	93 (24.3)	59 (45.7)	**< 0.001**
Leucopenia (%)	129 (5.7)	105 (27.4)	24 (18.6)	0.150
Elevated ALT (IU/L)	194 (37.9)	147 (38.4)	47 (36.4)	0.192
Elevated AST (IU/L)	209 (40.8)	157 (40.1)	52 (40.3)	**< 0.001**
Elevated ALP (IU/L)	138 (26.1)	103 (26.9)	35 (27.1)	0.057
Transaminitis	53 (34.0)	34 (8.9)	19 (14.7)	0.106
Prolonged PT&APTT (sec)	212 (41.4)	122 (31.9)	90 (67.7)	**0.001**
Hemoconcentration (%)	29 (5.7)	20 (5.2)	9 (6.1)	0.631

*PT* Prothrombin time, *APTT* Activated prothrombin time, *INR* International normalized ratio, *AKI* Acute kidney injury, *ALT* Alanine amino transferase, *AST* Aspartate aminotransferase, *ALP* Alkaline phosphatase

*Student *t* test or Mann-Whitney *u* test, where appropriate

**Fig. 2:**
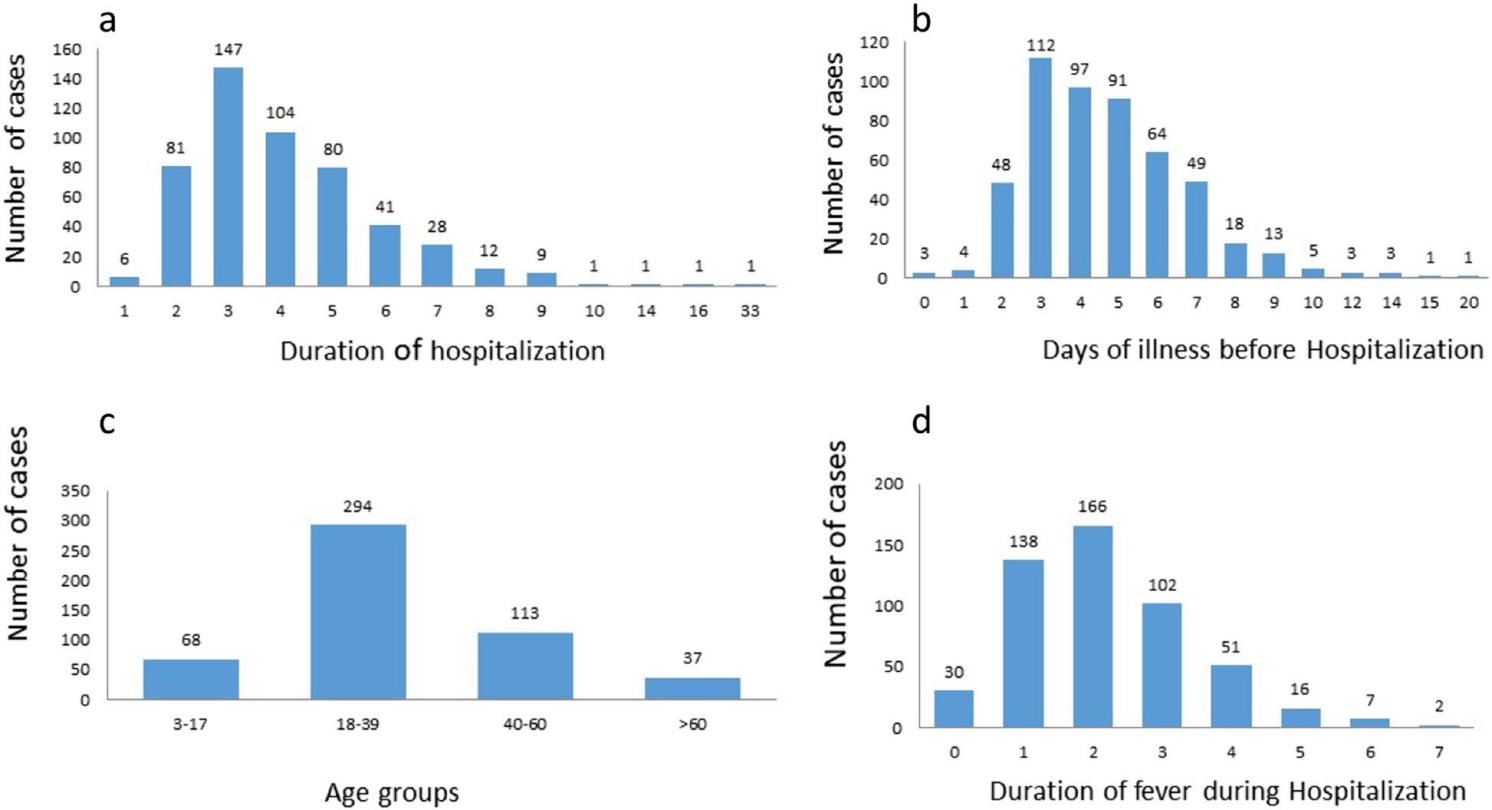
**a** Duration of hospitalization; **b** Days of illness before hospitalization; **c** Dengue cases in different age groups **d** duration of fever during hospitalization

Of clinical outcomes, prolonged hospitalization was more profound in DHF cases. The increase in duration of fever was observed in patients with DHF as compared to DF (Fig. 2). Similarly delayed hospitalization was observed more frequently in DHF patients than those with DF.

Univariate analysis (Table 4) revealed that shortness of breath (OR: 2.21), association with risk groups for dengue contraction (OR: 1.95), warning signs (OR: 2.18), delayed hospitalization (OR: 2.30), diabetes mellitus (OR: 2.71) and ascites (OR: 2.93) were significantly associated with DHF cases than DF cases. However, multivariate analysis revealed that delayed hospitalization, shortness of breath, diabetes mellitus, association with risk groups, and the presence of warning signs were associated with the development of DHF.

**Table 4:** Univariate and multivariate analysis for risk factors associated with DHF

Variables	Univariate analysis			Multivariate analysis		
	*P-value	OR	95% CI	*P-value	OR	95% CI
Age > 60years	0.089	1.82	0.91–3.64	---	----	---
Secondary Infection	0.512	0.491	0.06–4.12	---	---	---
Skin rash	0.265	0.77	0.53–1.19	---	---	---
Lethargy	**0.016**	1.69	1.10–2.58	0.21	1.40	0.83–2.37
Abdominal Pain	0.062	1.56	0.97–2.47	---	---	---
Thick gall bladder	**0.027**	0.472	0.24–0.92	0.99	0.43	1.41–3.75
Delayed hospitalization	**0.005**	0.57	0.38–0.83	**0.001**	2.30	1.14–2.78
Shortness of breath	**0.001**	0.42	0.26–0.70	**0.010**	2.21	1.20–4.05
Diabetes mellitus	**0.001**	0.329	0.18–0.62	**0.009**	2.71	1.29–5.69
Association with risk group	**< 0.001**	0.427	0.28–0.64	**0.007**	1.95	1.19–3.17
Warning signs	**< 0.001**	0.183	0.12–0.28	**< 0.001**	2.18	1.17-4.00
Residence	0.969	0.99	0.54–1.81	---	---	---
Liver cirrhosis	0.836	1.19	0.23–6.21	---	---	---
Jaundice	**0.048**	3.82	1.01–14.45	0.61	1.53	0.29–8.07
Sore throat	0.565	1.24	2.25–8.85	---	---	---
Convulsion	**0.026**	12.22	1.35–110.4	0.16	6.28	0.49–81.15
Ascites	**< 0.001**	4.46	2.25–8.85	**0.010**	2.93	1.28–6.67
Elevated AST	**0.030**	2.30	1.23–4.29	0.58	1.26	0.55–2.87

Odds ratio (*OR*) and Confidence interval (*CI*) have been rounded off

*Variables with *p* < 0.25 were excluded from multivariate analysis

The recovery of renal and hepatic functions was also evaluated at the time of discharge. The renal and hepatic insufficiencies were more prevalent among patients with DHF than those with DF.

## Discussion

This study is first of its kind to report a large cohort of dengue patients in Pakistan and evaluates clinico-laboratory features of DVI as well as risk factors of DHF. In Pakistan, dengue is currently the most debilitating and life-threatening viral infection. This study showed that patients with DF and DHF present with distinctive clinical and laboratory characteristics. Timely identification of risk factors of DHF will facilitate early identification and prioritized management of patients with severe dengue infections.

Comparing the demographics of DF and DHF revealed that males had a higher infection rate (69.53%) than females (30.64%). The prevalence of dengue cases might appear to be higher in males due to engage in outdoor activities. There could be the differences in the cultural or societal influences and immune response differences in the gender. However, the distribution of DHF occurrence was not statistically significant (Table 1). These finding are consistent with the results of other studies [22–25]. Study cohort demonstrated that the prevalence of DF (77.64%) was common in age group ≤ 40 year and prevalence of DHF (34.4%) was common in age group > 40 years. Age group distribution of DF and DHF is in accordance with the previous studies [22]. We reported the greatest number of cases in those under 40 years of age, with the highest incidence in those aged 19 to 39 years. This may be due to exposure with external environment for the purpose of education, business visits, provision of health care measures to the children and as an attendant of their elders, travel to endemic areas. These factors are linked with higher likelihood of contracting DVI among youth as compared to children, elders, and females of the same age groups, who have the least contact with external environment [22, 24, 26]. DVI is caused by weakened immunity, co-morbidities like diabetes, hypertension and secondary infection in advance age [27]. In advance age; changes in the cellular and humoral systems may account for a decline in immunity and alterations in clinical laboratory features [28].

In our study, fever was a common clinical manifestation in both DF and DHF patients, lasting between 1 and 7 days [29]. Long duration of fever was observed in DHF patients compared to DF (mean value 2.4 ± 1.4 versus 2.1 ± 2.1) (Table 2). In cases of DF, fever resolves on the third or fourth day after admission, whereas in cases of DHF, fever persists for more than 10 days. DHF cases have a longer duration of fever than DF cases, which indicates the severity of the disease [30]. It is important to note that prolonged fever is linked with delayed hospitalization, which attributes to the economic burden on health care system with limited health facilities, especially in developing nations such as Pakistan. In DHF, hospital stays lasted on an average of 4.7 days, while in DF, they lasted 3.19 days [31].

Existing evidence suggest that clinical manifestations are significant predictors of disease severity. They provide information regarding the disease severity, such as mild or classical dengue fever, dengue hemorrhagic fever, and dengue shock syndrome [22, 32, 33]. Other common clinical manifestations among dengue viral infection patients were myalgia, arthralgia, abdominal pain, nausea, vomiting, skin rashes, rigors and chills reported in literature. Similarly, hemorrhagic manifestations were more prevalent in patients with DHF than in patients with DF. These include bleeding from the gums and nose, petechia, purpura, and melena [29, 34], and similar findings were observed in our study.

There was high incidence of retro-orbital pain, jaundice, convulsions, coma, ascites and pleural effusion among the DHF cases than DF cases in current study (Table 2). In addition, Gingival bleeding and epistaxis are more prevalent in DHF than in DF (*P* = 0.006). Likewise, hematuria. Petechia and melena were significantly more prevalent in DHF (*P* = 0.001) (Table 2). These clinical manifestations and warning signs were excluded from the multivariate analysis because they are highly relevant to DHF, serve as diagnostic tools for the clinical case definition of DHF, and are consistent with previous research [35, 36]. Delayed hospitalization with DVI results in deterioration of clinical manifestations, which favors the progression of DF to DHF through the development of hemorrhagic manifestations.

Hematological profile is an additional indicator used to differentiate DF and DHF cases. The WBCs count was higher among patients with DHF than those with DF, but the difference was not statistically significant. Platelets count was significantly lower in DHF cases as compared to patients with DF. Thrombocytopenia was common laboratory finding in dengue viral infection but it was more prevailing in DHF patients in current study [35]. The hemorrhagic tendency increases because dengue virus affects the bone marrow and decreases the platelet cells production. Additionally, the virus can directly attack and destroy the platelets. Low platelet counts significantly increase the risk of bleeding [36]. The proportion of leukocytosis and raised HCT was significantly higher in DHF patients (Table 3). The normalization of plasma leakage was more in DHF due to intravenous rehydration intervention which has great impact on HCT. Hemoconcentration act as biomarker of plasma leakage and increased vascular permeability. The cut-off value for male and female are 47% and 40% respectively. The literature indicates a strong association between dengue fever and plasma leakage. The coagulation cascade is a series of events that occur in the body to form blood clots, which are critical in preventing excessive bleeding during dengue virus infection. The DVI disrupts the coagulation cascade, resulting in abnormal bleeding tendencies. Increased PT and aPTT levels (Table 3) are associated with the severity of dengue infection due to a disruption in the coagulation cascade. Elevation of both PT and aPTT was statistically significant in DHF (*p* 0.001) compared to DF, which is consistent with previous research [37, 38]. Hemorrhagic manifestations, shock and plasma leakage is due to the decreased levels of circulating protein C, S, antithrombin III and elevated levels of tissue factor, thrombomodulin. Similarly, reduction in coagulation such as II, V, VII, VIII, IX, X, antithrombin, and alpha-2 antiplasmin factors was reported in DHF [22, 35, 38].

Timely hospitalization is mandatory to avoid the severity of dengue infection. Delayed hospitalization and late diagnosis of dengue virus may worsen the infection and shift the DF to DHF and /or DSS. Our study showed that delayed hospitalization tends to lead the patient in severity of the disease and these results are in accordance with the previous studies [4, 39]. Increased in bleeding tendencies, delayed in hospitalization [39], hospital admission with warning signs [40, 41], co-morbidities [42] are all associated risk factors of DHF. These findings are harmonious with the reported studies [4, 31]. Additionally, certain co-morbidities like diabetes mellitus, hypertension, chronic kidney disease, allergic reactions, asthmatic reactions, ischemic heart disease and hepatic aberrations might place number of patients at high risk of developing DHF and DSS [32]. In DHF, liver cirrhosis (3.1%), hypertension (20.16%), and hyperlipidemia (3.86%) were additional concurrent co-morbidities (Table 1). Their incidence was considerably lower in DF. Statistically we found that there is strong association of diabetes mellitus (DM) with DHF while the patient suffering from DM individually had higher odds for developing DHF than patients without disease. Increased capillary fragility and permeability due to activation of T lymphocytes and release of cytokines in DM are might be some possible factors of development of DHF dengue [22, 42]. Secondary infection was associated with DHF cases more than DF (Table 4), but there was no statistical association between secondary infection and the development of DHF. According to WHO criteria, DHF patients exhibited increased capillary permeability or plasma leakage, which shifted towards DSS with signs of shortness of breath and circulatory failure [43]. Patients presents with SOBs had the higher odds (2.21) of developing the DHF. Warning signs at the time of admission will direct the clinicians to assess the presence of DHF and DSS [44].

The incidence of dengue viral infection was high in urban areas compared to rural areas. This could be due to the rapid urbanization from rural area and overcrowding. The other risks of contraction of DVI are living at dense population, living near stagnant water resources and poverty [45]. It will serve as carrier of DVI. These disease transmission risk factors have been endorsed by WHO [21]. Comorbidities such as hypertension, DM and CKD were more prevailing in DHF cases as compared with DF (Table 1). Among the disease transmission risk factors mosquito production with the stagnant water in the containers, discard tires flower pots, Mega structures or densely populated areas can harbor more breeding sites due to presence of water storage facilities, non-fogging areas where insecticide spraying is not done regularly and rapid urbanization and population density were predefined risk factors of DVI [4, 27, 46]. About 32.62% of studied population has association with the risk groups for contraction of dengue infection. The order of risk group association with dengue virus was living and travelling to dengue endemic areas > living near stagnant water > mosquito production site > non- fogging zone > mega structure > family history. The most alarming risk factor in our study was travelling and living in dengue endemic areas. Our findings were harmonious with the previously reported study [47]. Awareness of above-mentioned risk group helps to aid the anti-dengue programs to take the preventive measure in affected areas. Dengue infection is at peak from August to December due to the monsoon waves that promotes the heavy rainfall. This will provide the ideal situation for vector breeding. Fortunately, most of cases were reported at the time of epidemic included in study. During the months of epidemics; vector control measure must be in full swing in order to combat the disease and for the safety of community [48–50].

Unusual manifestation of dengue fever was multi-organ involvement in most of the cases which may include liver, kidney, central nervous system and heart. Liver is the most targeted organ by dengue virus. Hepatic injury is more prominent in DHF and characterized by elevation in hepatic enzymes [51]. Patient with pre-existing hepatic disease such as hepatitis are more susceptible to hepatic injury during the course of DVI [52]. Hepatic involvement is suspected if patient complains nausea, vomiting diarrhea, abdominal pain and anorexia. The possible mechanism of hepatic injury is unknown but it was reported in literature from patient’s autopsy specimen that dengue virus interacts with hepatocytes and induces micro-vesicular and macro-vesicular steatosis and councilman bodies. It damages the kupffer cells but the major damage involves intracellular damage of hepatic cells via necrosis [52, 53]. T-lymphocytes activation is also considered one cause of hepatic cells damage in dengue infection [54]. The levels of AST, ALP and ALT were significantly higher among patients with DHF than DF. The levels of AST were significantly (*P* < 0.001) higher among patients with DHF and these findings are in agreement with the previous reported studies [53, 55].

Tropism nature of dengue virus enables it to invade cardiac fiber, brain tissue, type-II pneumocytes, monocytes, macrophages and lymphocyte. This tropism results in multi-organ failure (MOD), particularly among patients with severe dengue infection. Patients with MOD have concomitant abnormalities in two and/or more than two organs in critical illness/febrile illness. It is important to note that MOD is associated with significantly mortality among dengue cases [22]. In our study 75% fatal cases had MODs. AKI in three (3), renal complications in eight (8), hyponatremia in one (1), hyperkalemia in three (3), pulmonary complications in three (3), thrombocytopenia in eight (8), septicemia in three (3), DHF in two (2) and DSS in three (3), ECG abnormalities in four (4), metabolic acidosis in three (3), acute respiratory syndrome in one (1) and circulatory failure in two (2) cases were observed among all fatal cases. In four fatal cases, the average length of hospitalization was less than 4 days, while in the remaining fatal cases, the average length of hospitalization exceeded 4 days.

Although fatality is uncommon in dengue infection but severe dengue cases are linked with higher mortality rates [56]. In our study, fatal cases were associated with severe form of DVI with MODs [57]. Delayed hospitalization deteriorates the health status of patients and worsens the clinical presentation, and contributes to mortality [58]. Our study’s findings are consistent with those of other reports on mortality due to severe DVI [31, 57].

In our study, the management of patients was symptom-based. Patients were administered oral and/or intravenous hydration therapy, acetaminophen for fever, antihistamine, proton pump inhibitors, and antiemetics. Plasma transfusions were administered to 20 patients with DF and 16 patients with DHF. The majority of patients were fully recovered prior to discharge. Nonetheless, some of the patients had mild to severe renal and hepatic insufficiency at the time of discharge from the hospital (Table 5).

**Table 5:** Renal and hepatic anomalies among the dengue patient on discharge

Variable	Total cases (*N* = 512) *n* (%)	DF (*N* = 383) *n* (%)	DHF (*N* = 129) *n* (%)
**Renal insufficiency on discharge**			
Normal renal function	458 (89.5)	343 (89.6)	115 (89.1)
Abnormal renal function Scr < 200µmol/L	38 (7.4)	31 (8.1)	7 (5.4)
Abnormal renal function Scr > 200µmol/L	16 (3.1)	9 (2.3)	7 (5.4)
**Hepatic dysfunction on discharge**			
Normal hepatic function	383 (74.8)	308 (80.4)	75 (58.1)
Mild Transaminitis (> 2fold of normal value)	106 (20.8)	62 (16.2)	44 (34.1)
Moderate to severe Transaminitis (> 3 fold the normal value)	24 (4.5)	14 (3.4)	10 (7.8)

Being a retrospective study, some limitations should be considered while interpreting the results. All the reported values are based on documentation in patients` profiles. Secondly biasing in clinical outcomes may be due to lack of standardized dengue management techniques and protocols. Thirdly viral load could not be determined due to the retrospective nature of study. Furthermore, patients were not followed-up to assess full recovery. Risk factors associated with the mortality were not addressed due to few deaths cases that negatively influence statistical power of current study. Despite these limitations, this study is a comprehensive presentation of DVI, the distinguishing characteristics of DF and DHF, and the risk factors of severe forms of disease by employing a large data set.

## Conclusions

This study revealed that approximately one-quarter of patients with DVI exhibit DHF. Patients with DHF demonstrate with distinct clinical and laboratory spectrum as compared to those with DF. Our study showed delayed hospitalization, diabetes, hypertension, warning signs, shortness of breath and ascites as predictors of DHF. Early identification and comprehension of DHF/DSS development predictors would aid in stratification of high-risk patients. These findings will assist clinicians in reducing dengue-related morbidity and mortality through timely administration of appropriate treatment for severe cases. Our research will also aid dengue control authorities in designing and implementing dengue control and management guidelines.

## Data Availability

All data generated or analysed during this study are included in this article.
